# A Scoping Review of Existing Policy Instruments to Tackle Overweight and Obesity in India: Recommendations for a Social and Behaviour Change Communication Strategy

**DOI:** 10.12688/f1000research.149857.2

**Published:** 2024-11-18

**Authors:** Nishibha Thapliyal, Shalini Bassi, Deepika Bahl, Kavita Chauhan, Kathryn Backholer, Neena Bhatia, Suparna Ghosh-Jerath, Lopamudra Tripathy, Preetu Mishra, Seema Chandra, Monika Arora

**Affiliations:** 1 Health Promotion Division, Public Health Foundation of India, New Delhi, Delhi, 110030, India; 2 Institute for Health Transformation, Deakin University, Global Centre for Preventive Health and Nutrition, Geelong, Australia; 3 Food & Nutrition Department, Lady Irwin College, University of Delhi, New Delhi, 110001, India; 4 The George Institute for Global Health, New Delhi, 110025, India; 5 UNICEF India Country Office, New Delhi, 110003, India; 6 New York University, New York, New York, USA

**Keywords:** Obesity, overweight, children, adolescents, pregnant women, lactating women, overnutrition, nutrition, physical activity, double burden of malnutrition, social and behaviour change communication, programmes

## Abstract

**Background:**

The Indian government is committed to addressing various manifestations of malnutrition, including overweight and obesity, inorder to improve individual health and well-being. The scoping review aims to map existing national policy instruments (programmes, schemes, regulations and guidelines) addressing overweight and obesity in India and analysing them for Social and Behaviour Change Communication (SBCC) strategies.

**Methods:**

Systematic identification and selection of policy instruments using ‘Arksey and O’Malley’ framework was conducted from central government ministry websites, between March and June 2023. These instruments focused on nutrition and/or physical activity, targeting specific demographic groups like pregnant women, lactating mothers, children (0-5 years and 5-9 years), or adolescents (10-19 years); excluding those focusing on specific diseases like micronutrient deficiencies, wasting, and stunting. Based on search strategy six policy instruments were included and analysed for SBCC strategies.

**Results:**

While many policy instruments incorporated SBCC plans; the ‘National Programme for Prevention of Non-Communicable Diseases (NP-NCD)’ stands out as a significant policy initiative specifically targeting the prevention of overweight and obesity within the broader context of Non-Communicable Diseases. It adopts a comprehensive approach addressing key drivers contributing to overweight/obesity across multiple levels of behavioural influence i.e., individual, interpersonal, community and organisation for health promotion. However, there’s need to strengthen SBCC strategies related to prevention and management of obesity, especially screening and counselling, to cover all age groups with a particular focus on adolescents and youth. SBCC strategies can also be incorporated into India’s Integrated Nutrition Support Programme (POSHAN 2.0) and/or Reproductive, Maternal, New-born, Child, Adolescent Health and Nutrition (RMNCAH+N) under the National Health Mission.

**Conclusion:**

This paper underscores the necessity for comprehensive strategies to address multifaceted origins of overweight and obesity. The NP-NCD stands out as a noteworthy initiative, and there is considerable potential for other programmes to emulate SBCC strategies to bolster their overall effectiveness.

***Note***: *Policy instruments throughout the paper have been used to cover programmes, schemes, regulations and guidelines.

## Introduction

The co-existence of all forms of malnutrition including wasting, stunting, micronutrient deficiencies and overweight and obesity-known as the triple burden of malnutrition is prevalent in many low-middle-income countries, ^1^ including India. ^2^ An estimate from 129 countries shows, 57 countries are experiencing an existence of both ‘undernourished children’ and ‘overweight adults’. ^3^ In India, successive rounds of the ‘National Family Health Survey (NFHS)’ have revealed a double burden of malnutrition, across all age groups ^4^^,^^5^ and micronutrient deficiencies among children and adolescents (0-19 years). ^6^ This is worrisome, as all the critical stages of life get affected, including early childhood, adolescence, pregnancy and lactation. Childhood obesity is associated with a higher chance of premature morbidity, death and disability in adulthood. ^7^ Likewise, maternal obesity during pregnancy is associated with increased risks of adverse maternal, foetal, and childhood outcomes, ^8^ underscoring its significance as a public health concern. Maternal obesity has even been associated with decreased initiation of breastfeeding, reduced duration of breastfeeding, along inadequate milk supply compared with their normal weight counterparts. ^9^

The drivers of overweight and obesity occur at the individual level (age, gender, inadequate physical activity, unhealthy food habits, undernutrition in early life, nutrition literacy and increased screen time), ^10^^–^^14^ interpersonal level (paternal and maternal nutrition status, inadequate breastfeeding practices and early introduction to formula foods, ^15^ education status, household income, socio-economic status, obesogenic home environment), ^16^^–^^18^ community (obesogenic food environment, inadequate physical activity environment), ^18^^–^^20^ organisational level (obesogenic food environment, inadequate physical activity environment), ^18^^–^^20^ and at policy environments (policies that enable or constrain physical activity and healthy eating). This suggests that unhealthy dietary habits and lack of physical activity are not primarily attributed to individual choices, as they are influenced by multiple factors operating across these levels. ^11^ Therefore, to prevent overweight and obesity at all critical ages (children, adolescents, pregnant and lactating mothers), it is crucial to address factors operating across all levels of influence.

In the context of India, the diverse cultural norms exhibit significant regional variations. These patterns are deeply intertwined with lifestyle choices, like eating habits and physical activity which collectively determine energy balance and consequently our body weight. ^21^ Recognising the need to adopt healthier diets and being more physically active are behavioural challenges that are pivotal aspects of combating obesity. ^11^ The cost-effectiveness of Behaviour change communication techniques is reported to be higher for obesity prevention compared to their application in management and treatment. ^22^^,^^23^ Social and Behaviour Change Communication (SBCC) is a recent, yet widely recognized approach that emphasises improving health outcomes through strengthening social context, i.e. improving behaviours of individuals and groups by addressing key drivers that shape these behaviours across various levels of influence. ^24^ A systematic review has highlighted the effectiveness of SBCC in preventing and reducing stunting and anaemia, ^25^ indicating its potential in addressing various health risks. This approach has also been shown to be successful in tackling breastfeeding and complementary feeding, ^26^ indicating that SBCC can and does succeed in improving the uptake of those practices. While it is crucial to recognize the effectiveness of SBCC in addressing overweight and obesity in India, there is currently a lack of a scoping review that examines the role of SBCC within existing policy instruments aimed at tackling this issue. Understanding the extent to which current policies integrate SBCC approaches to address overweight and obesity in India is essential yet remains unexplored in the available literature. The insight from this review will be instrumental in guiding future actions, including programme evaluations to identify ‘what is working’ and ‘what is not ’, implementing mid-course corrections, and shaping policy recommendations to strengthen the integration of SBCC into health policies aimed at addressing overweight and obesity in India. Thus, to address this gap, we conceptualised a scoping review to: 1) map existing policy instruments, including national programmes, schemes, regulations and guidelines that address overweight and obesity in India to understand the extent of national action; 2) evaluate the extent and nature of SBCC strategies employed alongside these policy instruments and; 3) offer recommendations to prevent overweight and obesity in India using SBCC techniques.

## Methods

### Design

To map the current national policies that address overweight and obesity in India we adopted ‘Arksey and O’Malley’s’ five-stage scoping review framework. ^27^

*Identifying research question*

We started by identifying the broader purpose of examining the existing policies related to overweight and obesity, nutrition, and health in India, aiming to understand how SBCC are integrated within these policies. Moving forward, our focus shifted to assessing the extent of existing policies covering overweight and obesity only. This involved examining national programs, schemes, regulations and guidelines. Additionally, we sought to understand the methods, mechanisms and approaches to how SBCC strategies are incorporated alongside these policies. Based on these inquiries, we consolidated the data into a single comprehensive question, to align with the objectives of the scoping review. 

“(1) What is the extent of existing policy instruments, including national programmes, schemes, regulations, and guidelines, addressing overweight and obesity in India, and (2) how are SBCC strategies integrated alongside these policies?”

i. *Identifying relevant policies*

This step involved developing a comprehensive search strategy to systematically identify policies related to overweight, obesity, nutrition and health in India to capture both government documents and grey literature. The search was carried out by two researchers (NT, DB), and any discrepancies were further resolved with input from a third researcher (SB).

We initiated our search strategy by compiling a list of all central government ministry websites in India (n=49). In the absence of a centralized database for Indian government policies, we systematically searched each of these ministry websites for relevant information. Additionally, we searched grey literature from various sources (NGO publications, International agencies, and independent research organizations involved in health policy in India). This approach ensured that we captured both formal and informal policy instruments.

• This combined list was then shared with domain experts (n=5) in public health and policy development. Their input helped us review and refine the list to ensure that no relevant ministries were missed.
• From this defined set of ministries, we included all potential government ministry websites (n=7) relevant to health, diet, nutrition, and public policy.
• Subsequently, using the refined list of ministries, we systematically scanned their websites for policies including programmes, schemes, regulations and guidelines addressing the aspects of diet”, “nutrition”, “undernutrition”, “malnutrition”, “overnutrition”, “Double Burden of Malnutrition (DBM)”, “physical activity”, “overweight”, “obesity” in India.

iii. *Selection of Policies: Inclusion criteria*

In the next step, two researchers (NT and DB) systematically selected policy instruments based on predefined inclusion criteria.

**Table:** 

Inclusion Criteria	Exclusion criteria
Policy instruments that address obesity directly or indirectly either through a focus on nutrition and/or physical activity as a primary or secondary objective and targeted pregnant, lactating women, children (0-5 years and 5-9 years age groups), or adolescents (10-19 years) nationally.	Policy instruments with a focus on preventing or treating specific diseases, such as micronutrient deficiencies or wasting and stunting, were excluded.

**Figure 1.**
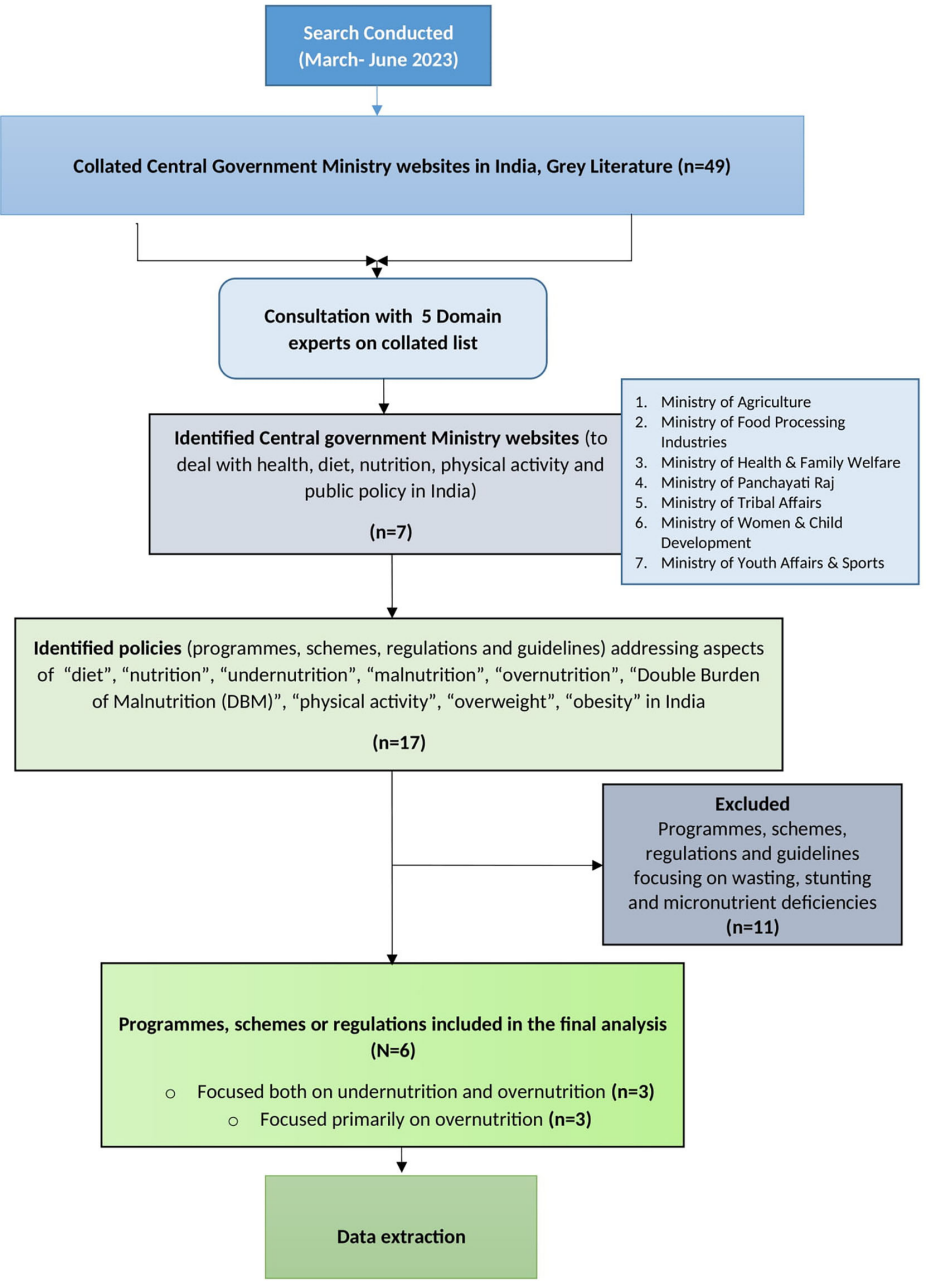
Brief study methodology.

The decision to exclude policies addressing micronutrient deficiencies and wasting/stunting was made to maintain a specific focus on overweight and obesity. While all forms of malnutrition are interconnected, their causes, interventions, and policies differ significantly, and including them would have diluted the review's focus. This decision was also guided by input from domain experts (n=5). The methodology is schematically outlined in Figure 1. The search was conducted by researchers between March and June 2023.

iv. *Collating, summarizing, and reporting results
*

Following the identification of relevant policy instruments, we collated and summarised findings from the review process. Seventeen policy instruments with a focus on malnutrition and targeting pregnant women, lactating women, children and adolescents (0-19 years) were identified (n=7). Out of the 17 identified policies, eleven were excluded as they addressed wasting, stunting and micronutrient deficiencies. The excluded policy instruments were ‘Integrated Child Development Scheme (ICDS), ^28^ Infant Milk Substitute, Feeding Bottles and Infant Foods (Regulation of Production, Supply and Distribution) Amendment Act, 2003 (IMS Act), ^29^ Janani Suraksha Yojana (JSY), ^30^ Scheme for Adolescent Girls, ^31^ Janani Shishu Suraksha Karyakram (JSSK), ^32^ National Food Security Act, ^33^ Rashtriya Bal Swasthya Karyakram (RBSK), ^34^ Mothers’ Absolute Affection (MAA) for Infant and Young Child feeding, ^35^ Maternity (Amendment) Benefit Act, ^36^ Poshan Abhiyan (National Nutrition Mission), ^37^ Pradhan Mantri Poshan Shakti Nirman’ scheme (PM POSHAN). ^38^ Six policy instruments were included in the analysis. The programmes (n=3) included in the analysis were Rashtriya Kishor Swasthya Karyakram (RKSK), ^39^ Eat Right India, ^40^ Ayushman Bharat [School Health and Wellness Programme and Pradhan Mantri Jan Arogya Yojana (PM JAY)], ^41^ that focused on all forms of malnutrition - wasting, stunting, micronutrient deficiencies and overweight-obesity; and remaining three (n=3) i.e. Fit India Initiative, ^42^ Food Safety and Standards (safe food and healthy diet for school children) Regulations, ^43^ and National Programme for Prevention and Control of Non-Communicable Diseases (NP-NCD) ^44^ focused on aspects of overweight-obesity ( Figure 1). The implementation of these programmes spanned from the year 1975-2023.

v. *Mapping of data*

For all policy instruments addressing overweight or obesity, we categorised their SBCC strategies using the ‘Socio-ecological model’ (SEM) framework, ^11^ to understand the type and levels of influence that the SBCC strategies may have on dietary and/or physical activity behaviours.

The SEM framework was chosen as it is a widely recognised and established framework for comprehending the intricate interplay between individual behaviour and the social and environmental factors that shape these behaviours. ^11^^,^^45^ It serves as a valuable tool for developing behaviour change interventions due to its ability to provide a profound understanding and insight into various levels of interactions between individuals, and their environment within a social system. This framework comprises five levels, namely, individual, interpersonal, community, organisational and policy environment. At the individual level, the framework reviewed how knowledge, attitudes, behaviours, and skills influence an individual’s actions. The interpersonal level focuses on an individual’s relationships with other people, such as family, friends, or peers. The community level explores strategies for connecting or reaching a broader audience. The organisation level looks at structures like schools, workplaces, cafes, restaurants etc. to support the adoption of rules and policies for adopting or inculcating healthy behaviours. ^11^ Similarly, the enabling policy environment focuses on approaches adopted by the government to address the needs of larger populations by introducing laws, policies, and legislations to encourage the adoption of healthy behaviours. Table 1 presents how each policy instrument addressed overweight or obesity and describes the SBCC strategies used, according to the SEM ( Table 1).

**Table 1.** Programmes, schemes, regulations and guidelines: Summary of Social and behavioural change communication (SBCC) strategies using Socio-Ecological Model.

Policy Instruments ^i^	Target Group	SBCC strategies as per SEM				
		Individual Level	Inter-personal level	Community level	Organisational Level	Policy environment
Rashtriya Kishor Swasthya Karyakram, 2014 ^39^	Adolescents (10-19 years)	✓	✓	✓	✓	✓
Ayushman Bharat/Pradhan Mantri Jan Arogya Yojna (PM JAY), 2018	Vulnerable urban and rural population, school children, pregnant and lactating mothers, at risk population School Health Programme- school going children	✓	✓	✓	✓	✓
Eat Right India, 2018 ^59^	All ages	✓	✓	✓	✓	✓
Fit India Movement, 2019 ^42^	All citizens with a focus on children and adolescents	✓	✓	✓	✓	✓
Food Safety and Standard Authority of India (FSSAI) (Safe foods and balanced diet for Children in School) Regulation, 2020 ^43^	School children, children in crèches	✗	✗	✗	✓	✓
National Programme for Prevention of Non-Communicable Diseases (NP-NCD), 2023 ^44^	All age groups (18 years and above for health promotion and 30 years and above for screening)	✓	✓	✓	✓	✗

✓: Strategies present at a particular level; ✗: Strategies absent at particular level.

## Result

1. *Existing policy instruments, including national programmes, schemes, regulations, and guidelines, addressing overweight and obesity in India*

We identified 17 policy instruments, of which eleven (n=11) were excluded. The remaining six were evaluated for their use of multifaceted interventions at different levels of influence, as outlined in the SEM framework. These programmes, including Rashtriya Kishor Sawathya Karyakram (RKSK) and Ayushman Bharat School Health and Wellness Programme, target adolescents, vulnerable populations and school children, employing SBCC strategies across all levels i.e. individual, interpersonal, community, organizational and policy levels. Other initiatives like the Eat Right India, which focuses on educatingfood safety and nutrition, and the Fit India movement, which promotes physical activity, engage citizens of all ages, while the NP-NCD focuses on health promotion and the screening & management of adults, though it lacks policy-level SBCC strategies.

2. *Integration of SBCC strategies alongside identified policies*

SBCC actions were integrated at different SEM levels, into the identified policies (n=6) as follows.

### Rashtriya Kishor Swasthya Karyakram (RKSK), 2014

The “Rashtriya Kishor Swasthya Karyakram (RKSK)” launched in 2014, aims to provide a holistic continuum of care to adolescent girls and boys with emphasis on marginalised and underserved groups. The programme encompasses six priority areas including nutrition. The behavioural strategies of the programme target adolescents in schools, families and communities through multiple strategies using outreach by counsellors, facility-based counselling, and strengthening of adolescent-friendly health clinics across levels of care. ^39^ SBCC activities were identified across all levels of the SEM under RKSK.

*Individual level:* Adolescent Friendly Health Clinics (AFHCs), ^46^ are to be established for providing individual counselling to adolescents, on various adolescent health issues including diet, with help from trained counsellors, Auxiliary Nurse Midwifery (ANMs) and Medical Officers at Government Medical Colleges, District Hospitals, Community Health Centres, and Primary Health Clinics. Counselling aims to improve adolescents’ knowledge and adoption of healthy behaviours (including diet and physical activity), dissemination of pamphlets, handouts and display of posters at AFHCs. These clinics need to be established close to adolescents to enhance access to services, thereby increasing uptake and demand through the dissemination of messages and materials on behaviour change communication. ^46^

*Interpersonal level:* Interpersonal communication with adolescents at the village level is enabled with the help of Peer Educators (PEs). ^46^ Weekly participatory sessions with adolescent groups (comprising of 15-20 adolescents per group) are to be conducted (including with out-of-school adolescents). In addition, Adolescent Friendly Club meetings, comprising 10-15 PEs are to be organised monthly to discuss and clarify adolescents’ health issues with ANM. ^46^ The PEs are trained for six days and during this training one entire session focuses on diet and physical activity. ^47^ A means of interpersonal communication channel, which involves referrals between various centres, de-addiction centres and Non-Communicable Disease Clinics (NCD-clinics) (providing counselling to adolescents and their family members on healthy lifestyle) is recommended to be established to connect AFHCs with ‘school going’ adolescents and referrals for ‘out-of-school’ adolescents through Rashtriya Bal Swasthya Karyakram). ^48^

*Community level:* Mass communication using hoardings, banners, posters (at AFHCs, community, and schools), social media, telecom-based platforms (Saathiya Salah app ^49^) and helplines are to be conducted to maximise programme reach. At the community level, institutional approaches include involving teachers, counsellors, PEs and representatives of non-governmental organisations (NGOs) in promoting adolescent health and wellness. As per RKSK operational guidelines, Adolescent Health and Wellness Days (AHWDs) need to be organised quarterly at the village level where health check-ups of adolescents and many infotainment activities like plays, *nritya natak* (dance drama) etc. are to be conducted. AHWDs aims to generate awareness among adolescents, parents and the community on adolescent health issues including nutrition and prevention of NCDs. ^50^ Information and services including delivery of nutrient supplements i.e. prophylactic dose of Iron Folic Acid tablets and Albendazole for deworming; health screening including clinical testing and screening for anaemia and assessing Body Mass Index for adolescents; counselling on nutrition and balanced diet, cooking using local, seasonal produce are recommended for adolescents in the community during AHWDs. ^50^

*Organisational level:* Under the programme, schools are initiated to play a paramount role in becoming centres of health advocacy and delivery. Peer Education sessions are recommended to be implemented in schools with nodal teachers responsible for facilitating and monitoring. Community Health Centres/District Hospitals are expected to organise and conduct outreach services for adolescents and their parents, potentially at schools and colleges. The Adolescent Education Programme is expected to cover all secondary and senior secondary schools in the country, with trained teachers to provide health education on NCDs, nutrition, health and education, violence, substance abuse, etc. in schools. ^51^

*Enabling environment/policy level:* A National Adolescent Health Strategy has been developed to guide the programme with nutrition as one of the six identified themes (Reproductive and Sexual Health, Nutrition, Mental Health, Injuries and Violence including Domestic and Gender-Based Violence, Substance Misuse, Non-Communicable Disease) under the RKSK. ^52^ It emphasises a holistic and comprehensive model based on a continuum-of-care approach, implemented in collaboration with relevant ministries and state governments.

### Ayushman Bharat Yojana, 2018

Ayushman Bharat Yojana or the National Health Protection Scheme is a flagship programme launched by the Government of India in 2018. ^53^ It aims to provide healthcare services and insurance coverage to every individual, especially the poor and vulnerable. It provides a defined benefit cover of Rs. 5 lakhs (Rs 0.5 million) per family, per year, and aims to create a comprehensive network of healthcare delivery (especially primary healthcare services) and wellness infrastructure across the country. Until 29 December 2022, 0.15 million ‘Health and Wellness Centres’ have been operationalised to provide reproductive, maternal and child health services along with drug distribution, promotion of balanced, healthy diets and regular exercise, yoga and the usage of digital technology to provide a continuum of care. ^54^

The School Health and Wellness Programme (SHWP) under Ayushman Bharat was also launched in 2018 to strengthen the preventive and promotive aspects through health promotion activities. Under the School Health and Wellness Programme, a curriculum on nutrition has been included in the year 2018, and nutrition health education is provided to school children to influence their behaviour regarding health and wellness. ^55^ We have considered SBCC activities under both, Ayushman Bharat and the School Health and Wellness Programme for SEM analysis, as both include activities for promoting healthy weight.

*Individual level:* Home visits are to be conducted by Accredited Social Health Activist (ASHA) workers (female) and Multipurpose Workers (MPWs, male) to screen and counsel individuals on lifestyle modifications including adopting a healthy diet, engaging in regular exercise, and addressing communicable and NCDs. ASHA workers are trained to deliver health advice on diet and physical activity, as part of their curriculum. ^56^ Additionally, ANMs assist in taking measurements, such as Body Mass Index indicators, during Adolescent Health and Wellness days (AHWDs) to screen adolescents. ^50^

*Interpersonal level:* Family education and counselling are to be provided by ASHA workers, especially to pregnant women. Information provided to pregnant women covers nutritional requirements during pregnancy and for new mothers, information on early breastfeeding and complementary feeding practices. ^54^ Nutrition counselling for adolescents and women of reproductive age aims to addresses issues of low birth weight in new-borns and promotion of early initiation of breastfeeding to prevent childhood illnesses, prevention of anaemia, and consumption of iron folic acid supplements. The formation, handholding and functioning of patient support group (disease-based) meetings are also part of the programme. ^57^ These groups not only involve patients but also their family members. Family health folders are to be maintained by the ‘Health and Wellness Centre’ or nearby ‘Primary Health Centre’. Additionally, under the school health and wellness programme, two teachers in the school are to be trained to become ‘Health and Wellness Ambassadors’ for further imparting age-appropriate information sessions to students. These students can then act as ‘health and wellness messengers’ and engage in health promotional outreach activities to support their peers, parents and family. The programme also focuses on promoting yoga in schools among middle and high school children, particularly on International Yoga Day, through Health & Wellness Ambassadors. ^57^

*Community level:* The programme facilitates community engagement via community platforms such as Village Health and Nutrition Days (VHSND), Village Health, Sanitation, Nutrition Committees (VHSNCs), and the *Mahila Arogya Samities* (all women groups). These community groups and committees aim to generate awareness on locally relevant issues such as, lifestyle modifications for maintenance of a healthy diet and regular exercise to prevent cardiovascular diseases and diabetes. Additionally, the community platforms recommend organising periodic meetings and health camps to further promote health messages. Community Health Officers (CHOs) play an active role in health promotion, ensuring counselling, service support and monitoring of health promotional activities. ^54^ Under the RKSK, the Adolescent Health Days have been re-branded as ‘Adolescent Health and Wellness Days’ and ‘ *Yuva Samvad’* under Ayushman Bharat as Health and Wellness Centre (AB-HWCs). These events are scheduled quarterly with the involvement of primary healthcare teams from AB-HWCs. They serve as an edutainment platform to educate adolescents, families and other stakeholders on adolescent health issues including healthy eating, exercise and services available at AB-HWCs. ^50^

*Organisational level:* Under the programme, two teachers in every school are to be trained to become ‘Health and Wellness Ambassadors’. These “Health and Wellness Ambassadors” in schools are also designated to plan age-appropriate, skill-oriented, theme-based sessions for children who can further act as ‘Health and Wellness Messengers’. ^57^ Among the various themes for health promotion, two themes relate to overweight and obesity, specifically, ‘improving nutrition’ and ‘addressing conditions of NCDs ’. ^51^ Weekly, ‘Health and Wellness Days’ in schools are to be observed on Tuesdays. ^57^ Under the programme, counselling services at schools are to be made essential. ^50^

*Enabling Environment/Policy level:* The HWCs formed under ‘Ayushman Bharat’ are supported in the National Health Policy, 2017. ^58^ The policy recommends strengthening the delivery of Primary Health Care through the establishment of “HWCs” as the platform to deliver Comprehensive Primary Health Care.

### Eat Right India, 2018

The Food Safety and Standards Authority of India (FSSAI) as mandated in the preamble to the Food Safety and Standards Act, 2006, is tasked with ensuring the availability of safe and wholesome food for the Indian population. In pursuit of this objective, FSSAI initiated the ‘Eat Right India’ movement in 2018, aiming to revolutionize the nation’s food system to provide safe, healthy, and sustainable food for all citizens. Anchored by the tagline “Sahi Bhojan. Behtar Jeevan (Eat Right. Better Life),” this movement combines regulatory measures, educational campaigns, capacity-building initiatives, and collaborative efforts. Like other initiatives, Eat Right India endeavours to instigate behavioral change by addressing all levels of the Socio-Ecological Model. ^59^ Similar to other programmes, Eat Right India also intends to change behaviour by targeting all levels of Socio-Ecological Model.

*Individual level:* Education and awareness on food safety, nutrition (safe and sustainable diets) and hygiene is to be imparted among children in schools via the Eat Right school programme. ^60^ Content like a Yellow Book (age-appropriate books, in two volumes with each of them targeted at children from grade 1-8 to inculcate the right eating habits and are available in 11 languages), ^61^ a fun-filled activity book has been designed to teach and reinforce the message of safe and nutritious food, videos, ^62^ posters ^63^ and a food safety magic box (manual on easy tests for checking adulterants in food) ^64^ have been developed for integration into the school curriculum. ‘Health and Wellness Ambassadors’ (teachers) and teams (School Health Clubs) are to be made in schools to carry out Eat Right activities in schools. A teacher training manual was developed to give an in-depth understanding of the concepts of food safety and nutrition. ^61^

*Interpersonal level:* Parents are encouraged to be ‘health and wellness ambassadors’ by taking up an online certification course on food safety and healthy food by FSSAI, to sensitise others. ^65^

*Community level:* Community-based competitions like ‘low salt cooking competition’, quiz, ^66^ *mela* (fair), ^67^ challenges ^68^^,^^69^ and paint a wall are to be organised to spread positive messages. A mobile food testing van- ‘Food Safety on wheels’ ^70^ has been launched to access remote areas and conduct training and awareness activities. Eat right ‘tool kits’ have been developed to reach communities at the grassroots level. Education and awareness on food safety and nutrition are to be imparted to individuals in communities with the help of developed toolkits by frontline healthcare workers. ^40^ The toolkits are developed on two broad components - ‘Eat Healthy’ and ‘Eat Safe’. It attempts to deliver clear and simple messages on foods to eat (balanced diet, fortified foods, nutrition during the first 1,000 days) and foods to avoid (high fat, sugar and salt foods) and elimination of trans-fats from our diets. ^40^

Public Service Announcements (PSAs), videos, flyers, flipbooks, posters, social media and celebrities to be used to generate awareness among people to adopt dietary modification for healthy eating, for example – “ *Aaj Se Thoda Kam*” starring Indian celebrities to reduce HFSS food intake, ‘Trans-Fat Free India@75’ and ‘Heart Attack Rewind (against the use of trans-fat) and promotion of food fortification. ^71^ Furthermore, mascots ‘Miss and Master *Sehat*’ ^72^ to be utilized to spread awareness on safe and nutritious food in community. ^73^ Awareness campaigns on the importance of food fortification (adding important vitamins and minerals to food items) are to be implemented. ^74^

*Organisational level:* Many schools, colleges, workplaces, hospitals, etc. are encouraged to become a part of ‘The Eat Right Campus’ ^75^ initiative to promote safe, healthy and sustainable food on campuses across the country. Mascots ‘Miss and Master *Sehat*’ ^72^ are used to spread awareness on safe and nutritious food in education institutes. ^76^^,^^77^

*Enabling Environment/Policy level:* There are many regulations passed under the Eat Right India initiative, for example, the Repurpose Used Cooking Oil (RUCO) ^78^ that requires all *Food* Business Operators (FBOs) to monitor the quality of oil during frying (the limit for Total Polar Compounds at 25 percent beyond which the vegetable oil) by complying with the regulations from 1st July 2018 onwards; Packaging regulations(2019) ^79^ that provide guidelines for businesses looking to verify packaging materials and processes that are safe for food have been passed. ^80^ Voluntary public commitments made by individual chefs, bakeries and their associations are encouraged to commit to eliminating industrial trans-fat from their products to safeguard the health of the Indian citizens and provide them with healthy food environments. ^81^

### Fit India Movement, 2019

The Fit India Movement was launched in August 2019 by the Indian government to make fitness an integral part of our lifestyles by promoting physical activity. ^42^ It is a nationwide campaign with activities suited for people of all age groups. This initiative is comprehensive with SBCC strategies at all levels of SEM.

*Individual level:* A mobile application called ‘Fit India App’, ^82^ has been launched by the Government of India for all individuals to enable fitness and diet monitoring. Through this Mobile App, citizens can assess their fitness through a series of simple tests and are provided with regular information on how to improve their fitness levels. Additionally, the Fit India Mobile App has features such as setting daily activity and fitness goals and trackers to monitor daily activity, water intake, calorie intake and sleep.

*Inter-personal level:* Fit India Movement encourages “ **Youth Clubs”** to motivate and harness youth to become part of **Fit India Movement, to create mass awareness on the importance of physical activity**. Fit India Youth Clubs are a registered group of people who are aware of the importance of physical activity and who can motivate fellow citizens to get involved in 30-60 minutes of physical activity per day for five days a week. Youth clubs are motivated to inculcate approximately 60 minutes of physical activity in their daily plans, five days in a week.

*Community level:* As a part of the Fit India Movement, positive messages through dialogues and conversations on nutrition, and wellness with prominent sports personalities, health icons, influences, and health ambassadors are aired via various mediums (hoardings, banners, posters, television advertisements, social media- Instagram/YouTube etc) to motivate people to implement activities like the “Fit India Freedom Run’ ^83^ and, ‘Fit India Cyclothon’. At the community level, the Fit India Freedom Run, Plog Run (the activity of picking up trash while jogging), Cyclothon, and cycle rallies are to be organised.

*Organizational level:* Under the ‘Fit India school campaign’, ^84^ schools are encouraged to apply for Fit India School Certification which necessitates having atleast one teacher trained in Physical education, having a playground where two or more outdoor games can be played, having atleast one physical education period each day. It also necessitated having a physical activity hour (minimum of 60 minutes per day) for every child in schools. ^85^ The school is provided with certification when they satisfy the physical activity criteria laid down by the government. Parents, staff and school management committee members are encouraged to actively participate in club activities to act as role models for students. ‘Fit India School Week’ ^86^ is observed in all enrolled schools to promote messages through competitions like poster-making, poetry recitation, essay competitions, graffiti competitions, lectures and cultural events on topics related to physical activity and healthy eating. Schools are encouraged to celebrate ‘Yoga day’, ‘fitness day’, ‘pledge of fitness’ and ‘annual day during sports week culmination’. Workplaces are also included in the Fit India Movement, with businesses encouraged to organise weekly sports activities and open gym facilities for their employees at their workplaces. ‘Fit India Prabhat Pheri’ (morning walks) ^83^ by small as well as larger organisations like Resident Welfare Associations, Village, Town or City Council/Panchayat/NGOs are encouraged.

*Enabling policy environment:* Under the Fit India movement, the government has mandated a physical education period per day for every child in public and private schools under the Central Board of Secondary Education. ^87^ Fit India protocols have also been generated for all citizens of different age groups. ^88^

### Food Safety and Standards (Safe Foods and Healthy Diets for Children in School) Regulation, 2020

The Food Safety and Standards (Safe Foods and Healthy Diets for Children in School) Regulation applies to schools and crèches, and focuses on the provision of safe and healthy food to children and adolescents (> 22 months - 18 years). ^43^ According to our analysis, there are no SBCC strategies embedded in this regulations that cater to individual, interpersonal and community levels but concurrently, Organisational and Enabling environment/Policy levels exist as per SEM.

*Organisational level:* The regulation states that 75%-80% of the food available in school canteens should belong to the ‘healthy food groups’ i.e. cereal, millets and pulses, milk, milk products, egg, meat and fish, fruits and vegetables, oils, fats, nuts and oilseeds, processed/cooked foods. HFSS foods should not be made available or advertised on school campuses and areas within fifty metres from the school gate. ^43^ Schools are encouraged to engage with nutritionists to draft healthy menus for children. In addition to a well-balanced menu with a variety of foods in the school cafeteria, food labelling and display of healthy food options are encouraged under the regulation. The regulation necessitates, the school authority to ensure that signage boards containing the warning “Do not sell including free sale or market or advertise the food products high in saturated fat or trans-fat or added sugar or sodium within school premises or campus” are prominently displayed at the school gate. ^43^

*Enabling environment/policy level:* The regulation states meal times in schools to mandatory inculcate healthy eating habits in children. Under the regulation, guidelines, related to permitted foods and beverages and the quantity (to promote the use of cooking oil in moderate amounts) and quality (e.g., use of re-heated fats and oils shall be avoided) are issued. FBOs manufacturing are also barred from advertising and marketing of HFSS foods and beverages to children on school premises, including through logos, brand names, posters, textbook covers or in an area within fifty meters from the school gate in any direction. Sponsorships of sporting events in schools by FBOs can only be done if their foods are not HFSS. ^43^

### National Programme for Prevention and Control of Non-Communicable Diseases (NP-NCD), 2023-2030

NP-NCD, also previously known as the National Programme for Prevention and Control of Cancer, Diabetes, Cardiovascular Disease and Stroke (NPCDCS) ^89^ was launched in 2010 in 21 districts and expanded pan India in 2017. ^90^ It focuses on strengthening infrastructure, human resource development, health promotion (especially for 18 years and above), early diagnosis, management and referral, especially for the population aged 30 years and above. The programme offers a range of services including health promotion, psychosocial counselling, management (out-and-in-patient), day-care services, home-based care and palliative care as well as referrals at various health facilities. It is also the first programme that talks about the need for health education programmes that promote exercise, weight reduction, early diagnosis, screening and management of individuals with NCDs while addressing their risk factors at health facilities. ^44^ The programme targets the majority of SEM levels i.e. individual, inter-personal, community and organisational.

*Individual level:* Under the programme, the community health workers (ASHA/MPH/ANM) are to impart health education on healthier lifestyle behaviours like healthy eating and physical activity to individuals. Opportunist screening of individuals (above the age of 30 years) to identify individuals at risk of NCD is on basis of Community Based Assessment Checklist (CBAC) ^91^ which encompasses waist circumference measurement along with other at risk parameters of NCD by community health workers at PHCs. ASHAs are to capture enumeration and CBAC for all individuals above the age of 30 years at the household level during house visits. An NCD application (CPHC-HWC App) ^92^ has been developed for enumeration and to perform risk analysis of the enumerated individuals. ^93^ The appointed counsellor at Community Health Centres (CHCs)/PHCs, Sub-Centres and District Hospital (DH), are expected to provide counselling to the patients (>30 years) on increased intake of healthy foods, increased physical activity through sports, exercise, etc., at the time of their visit to a health facility. ^44^

*Inter-personal level:* Counselling of patient’s attendants/family members, who come along with the patient on healthy lifestyle (healthy diet, physical activity, salt reduction, avoidance of alcohol and tobacco) is to be carried out along with the patient to bring about effective inter-personal communication. The frontline community health workers are also to provide family-centric care and education on the adoption of healthier lifestyles and treatment under NP-NCD. ^44^

*Community level:* Camps are to be organised in the village, on VHSNDs when the health worker goes to the village for immunization and other health services. During these camps/designated days, health workers are expected to discuss the various aspects of a healthy lifestyle and its benefits with the target groups and motivate them to adopt a healthy lifestyle and practice regularly for the prevention of common NCDs. Key messages that need to be conveyed to the public include 1. increased intake of healthy nutritious foods 2. Increased physical activity through sports, exercise etc. During the camps/designated day, ANM and (or) Male Health Workers are expected to record the history of persons at and above the age of 30 years for physical activity and Body Mass Index etc. (via CBAC- opportunistic screening) and provide referrals to at-risk individuals. Opportunistic screening for NCD including waist circumference under CBAC forms is also required by the health workers. ^94^ Yoga is also an integral part of the programme. Under the programme, observance of NCD Week/Fortnight (Swasth Nagrik Saptah/Pakhwara) is also suggested to be organised as a platform to increase awareness regarding NCD and campaigns like K4H-Knowledge for Health for NCDs.

Health promotion on a healthy lifestyle (healthy diet, physical activity, salt reduction, avoidance of alcohol and tobacco) is to be carried out in the community. Locally prevalent folk media (street plays, wall paintings, hoardings etc.) is recommended to be used to reach the targeted population, particularly in rural and urban deprived populations.

The programme talks about using mass media for health promotion. Public awareness using the most effective channels (Radio, Television, Internet and Print media-pamphlets and hand-outs) that have reach the community are recommended to be used for health promotion. There is also a plan to utilise m-Health strategies for expanding access to health information on NCDs. m-Diabetes and m-Cessation interventions have been integrated for health promotion. ^44^

*Organisational level:* Women self-help groups/social groups and NGOs are recommended to be used for health advocacy. Health promotion activities are to be carried in schools, workplaces, during VHSNCs, Mahila Aarogya Samiti and Jan Aarogya Samiti etc. under the programme. ^44^

## Discussion

This paper investigates the SBCC strategies employed by the existing policy instruments in addressing overweight and obesity in India among children, adolescents, pregnant and lactating women. The outcomes of our study are significant as the rising obesity rate poses a considerable public health issue in India ^95^^,^^96^ and are recognised as major contributor to disability and death, globally. ^97^ The findings also seek to provide evidence for future actions by identifying gaps and recommend mid-course corrections, if needed, in the currently implemented programs.

Our scoping review revealed that before the year 2023, none of the policy instruments notably addressed the rapidly rising burden of overweight and obesity across various age groups (including pregnant women, lactating women, children and adolescents). However, the introduction of NP-NCD in 2023 marked a pivotal shift in our country’s approach to tackling obesity, as it recognises the intricate linkages between obesity and NCDs and underscores the significance of addressing obesity through a behavioural change approach. Among the six identified policy instruments, the recently revised NP-NCD programme ^44^ has the potential to serve as a comprehensive approach to tackling the escalating burden of obesity within the broader context of NCDs.

The growing prevalence of overweight and obesity across all age groups has led to an increased focus on implementing triple-duty actions, regulating the advertisement of HFSS foods, necessitating changes in governance, financial strategies, and capacity building. ^98^ The Lancet Commission on the global syndemic of obesity, undernutrition, and climate change also highlights the need to concurrently address these interconnected problems. ^99^ Considering the triple burden of malnutrition, and aligning with Target 2.2 of the Sustainable Development Goals, which aims to “end malnutrition in all its forms”, ^100^ the Indian government has taken applaudable steps to confront this emerging issue, by formulating the ‘Multisectoral Action Plan for Prevention of Non-Communicable Diseases’ ^101^ (2017-2022) with a special emphasis on prioritizing obesity prevention. This approach aligns with efforts in other countries like Ethiopia, Tanzania, United Kingdom, Kingdom of the Netherlands, where SBCC strategy action plans are tailored to health and nutrition ^102^^–^^105^ and Tonga for obesity prevention. ^103^ The revised NP-NDC programme acknowledges the interconnectedness of various biological and environmental health determinants, emphasising the importance of maximising the impact of interventions.

In the future, in the short term, apart from the NP-NCD programme, enhancing the integration of SBCC strategies into other existing national and sub-national level initiatives could be prioritised. Given the evolving landscape of malnutrition within the country, transitioning from stunting and wasting to the triple burden of malnutrition; it is imperative to incorporate triple-duty SBCC messages, campaigns, and interventions related to diet, services, and caregiver practices into programmes initially intended to address only stunting and wasting.

Furthermore, double duty action could be integrated into India’s recently launched Integrated Nutrition Support Programme i.e. Saksham Anganwadi and POSHAN 2.0. ^106^ This initiative could incorporate an exclusive plan to address overweight and obesity alongside efforts to combat malnutrition. POSHAN 2.0 presents an opportune platform, as it targets the strategic age group and seeks to develop and promote practices that nurture health, nutrition awareness and good eating habits for sustainable health, wellness and immunity in the community. ^106^

In the long term, allocating resources, both in terms of time and budget, for a national campaign targeting overweight and obesity is recommended. This initiative could entail partnering with leading communication firms known for their successful marketing strategies, extending even to remote areas. The existing NCD Divisions at the national, state and district levels that focus on the promotion of healthy diets and practices among the population under NP-NCD could consider the development of overweight-obesity guidelines including a focus on healthy eating practices, toolkits etc. for the population. While certain existing policy instruments have included this to some extent, there is room for more systematic and wide-reaching efforts so that all forms of malnutrition are brought to the same level of concern and attention as stunting, wasting and micronutrient deficiencies in the public sphere. This can be achieved by addressing its various underlying causes, with a particular focus on caregivers (families, parents especially mothers) and communities at large. Actions and strategies need to be ‘well-resourced’ at all levels by improving the provision of resources (both human and monetary). These campaigns should be developed based on an assessment of the prevailing behavioural patterns in the country, along with a thorough understanding of the underlying reasons for the same. Additionally, it is important to allocate dedicated budgets for SBCC activities. Providing definitive budget commitments, clear and realistic time-bound strategies, along with robust monitoring and evaluation frameworks can contribute to the efficacy and sustainability of the activities.

### Initiatives at different levels of SEM

SBCC strategies must be supported by actions at the policy level to create an enabling policy environment. For example, the effectiveness of SBCC strategies can be strengthened through comprehensive actions that provide both i.e., enforcement of SBBC strategies and mandates that lead to environment improvements which nurture behaviours. ^107^^–^^109^ For the matter, a commendable action taken in India has been the FSSAI, MOHFW ban on the sale and marketing of HFSS foods within schools and a 50-metres radius of the schools’ perimeter. ^110^ Such a policy can augment nutrition related SBCC strategies within schools by promoting an environment in schools where it is easier for students to put into practice what they learn. Another potential enabling policy for SBCC strategies could be subsidising healthy foods and increasing taxation on HFSS, as currently, healthy food options are more expensive than unhealthy foods in India. ^111^ A study conducted in seven countries found that subsidies for healthier food lead to a significant increase in the purchase and consumption of healthier food options. ^112^ There is a need to modify food labelling norms in India, for example, easy-to-understand front-of-pack labelling could potentially improve consumers’ food choices. ^113^ Mandatory front-of-pack labelling, easy-to-read labels (descriptive labelling) or health warnings on labels need to be introduced by the relevant authorities. Additionally, initiatives to empower the consumer to identify and understand these labels correctly need to be conducted to promote behavioural changes. ^114^ Other possible measures include reducing serving size, ^115^ plate size in restaurants, serving healthier items as welcome snacks and drinks, default vegetable and fruit in combination meals in fast food places, monitoring the quality and quantity of cooking oil in dishes at fast food chains and restaurants, similar to the FSSAI’s RUCO ^78^ initiative for small FBOs.

Transitioning to the organisational level, the Government of India has made commendable efforts to address overweight and obesity through initiatives such as the School Health and Wellness programme ^57^ under Ayushman Bharat. The programme aims to inculcate appropriate health and nutrition behaviours in school children. ^57^ Implementing simple efforts like having a fruit and vegetable policy in schools for a food break, significantly increases fruit and vegetable consumption in children. ^116^ *Poshan Vatika’s or nutri-*kitchens (community kitchen gardens), as planned under POSHAN 2.0, aim to encourage the use of locally available products to avoid malnutrition. A similar approach can be adopted in Indian schools through meaningful engagement of students. An increased intake of vegetables among students involved in creating schoolyard gardens was evident from a study conducted in the United States. ^117^ Descriptive labelling and attractive packaging of healthy food items can also be implemented in Indian schools to support behaviour change towards the consumption of healthy foods in school cafeteria’s. ^118^ Furthermore, promoting the availability of healthy eating options at public places ^119^^–^^121^ like cinemas, cafeterias, and gyms, can have a significant impact on an individual’s food choices, especially in the Indian context to promote the consumption of healthy eating among all age groups. Simultaneously, there is a need to provide physical activity infrastructure in schools, colleges and workplaces and other organisational settings to facilitate opportunities for increasing physical activity levels among children, adolescents, youth and adults. ^122^ My Plate for the Day’, developed by the Indian Council of Medical Research-National Institute of Nutrition (ICMR-NIN), MoHFW is designed on the basis of ‘Dietary Guidelines for Indians’ and ‘Nutrient Requirements for Indians’ to encourage and promote healthy dietary practices among the community. ^123^ The model plate is aimed to provide dietary diversity with a proper balance in macro- and micro-nutrients for a healthy individual of any gender. ^124^

We identified numerous initiatives undertaken by the Government of India to promote healthy lifestyles, especially physical activity among citizens at the community level like, the Fit India Movement, ^125^ community and individual counselling under NP-NCD, observing International Yoga Day, National Youth Day ^126^ and International Day of the Adolescents. These initiatives demonstrate the importance of a strong political commitment to prioritizing essential preventive health strategies for the population at large. Within these programmes, various communication tools such as flyers, infographics, health cards, cooking demonstration sessions, digital resources and discussion groups must have messages that are context-specific, non-technical, and delivered in local dialects. To promote physical activity, it is essential to enhance accessibility and infrastructural modifications in spaces such as public parks to encourage physical activity for people of all age groups.

The use of digital approaches to healthcare and prevention is increasing, particularly in low-middle-income countries. ^127^^,^^128^ The use of mobile health tools ^129^ to educate people in remote and hard-to-reach areas about nutritional counselling and physical activity has already been planned under the NP-NDC. However, the programme can significantly enhance its reach and effectiveness by targeting all community members by using new innovative social media activities like reels, WhatsApp messages, health quiz challenges, storytelling, interactive sessions etc. Social marketing can be integrated into multi-level ecological approaches, utilising multiple “P” (Price, Place, Product and Promotion) intervention strategies to support environmental changes that promote healthy behaviours in children. ^130^ Health promotion messages in-between advertisements can be developed and broadcasted in programmes and channels with children shows, as in the ‘CNN-BBC network joint campaign on health promotion messaging on coronavirus”. ^131^ Nutritional counselling of parents using m-health technology can also be provided. This approach is supported by the success of an Australian childhood obesity prevention internet-based programme for parents, which demonstrated improved dietary practices in children due to parental e-health counselling. ^132^ Technology can also be utilised for screening children for obesity in schools and institutions, aiding early identification and promoting overall health and well-being. ^133^

Moving forward, there is a critical need for robust research guided by Behavioural Insight Unit of NITI Aayog, the apex public policy think tank of the Government of India. ^134^ This research aims to gain better insight into drivers of obesogenic behaviours and identify potential nudges that can influence various segments of the population, including, children, adolescents, pregnant and lactating mothers. It is essential to understand the determinants specific to both rural and urban areas. Such research endeavours planned under the NP-NCD programme, would significantly contribute to building substantial evidence in combating overweight and obesity. However, it is noteworthy that scoping reviews provide a broad overview and are susceptible to bias and thus future research could take up more in-depth research, especially on grassroots implementation of planned SBCC strategies under programmes.

## Conclusion

The scoping review underscores the immense potential of India’s NP-NCD programme in addressing the formidable challenge of NCD, while also emphasising overweight and obesity prevention, through its comprehensive and multi-disease approach. The programme has the potential to influence behaviours and shape societal norms to promote healthier lifestyles.

The findings indicate that while many policies and programmes incorporate SBCC elements, the NP-NCD is particularly noteworthy for its targeted approach to preventing overweight and obesity within the broader context of NCDs. Its comprehensive framework addresses key drivers of overweight and obesity across multiple levels of behavioral influence, which is vital for effective health promotion. The success of NP_NCD would hinge on continuous and rigorous enforcement across all its components, as well as the development of supportive policies, regulations and an environment that foster healthy behaviour in the community. A focused approach towards adolescents and youth is essential, given their increased vulnerability to these issues. Furthermore, integrating SBCC strategies into existing initiatives like POSHAN 2.0 and RMNCAH+N under the National Health Mission could strengthen the overall effectiveness of health and nutrition programs.

## Authors’ contribution

MA, SB and DB conceptualized the study. NT, DB and SB contributed to scoping review, analysis and interpretation of results. NT, DB, SB, KC drafted the manuscript. KB, NB, SG, SC, LT, PM, PM, MA reviewed the manuscript critically for intellectual content. All authors reviewed and approved the final manuscript.

## Ethics and consent

Ethical approval and consent were not required.

## Data Availability

No data are associated with this article.
