# Supplementary material for: A Scoping Review of Existing Policy Instruments to Tackle Overweight and Obesity in India: Recommendations for a Social and Behaviour Change Communication Strategy
Source: F1000Res. 2024 Nov 18;13:496. Originally published 2024 May 17. [Version 2] doi: 10.12688/f1000research.149857.2 (PMC11759891; doi:10.12688/f1000research.149857.2)
Supplement: Peer review history

# Reviewer report: Reviewer response for version 2

**DOI:** 10.5256/f1000research.174399.r341411

**Published:** 2025-01-24

**Authors:** Palak Gupta

**Affiliations:** 1 Utah State University, Logan, UT, USA

Thank you for making edits based on my comments. The article looks good to me! Congratulations!

Are the rationale for, and objectives of, the Systematic Review clearly stated?

Yes

Is the statistical analysis and its interpretation appropriate?

Partly

If this is a Living Systematic Review, is the ‘living’ method appropriate and is the search schedule clearly defined and justified? (‘Living Systematic Review’ or a variation of this term should be included in the title.)

Not applicable

Are sufficient details of the methods and analysis provided to allow replication by others?

Partly

Are the conclusions drawn adequately supported by the results presented in the review?

Partly

Reviewer Expertise:

Food insecurity, hunger, Nutrition, public health

I confirm that I have read this submission and believe that I have an appropriate level of expertise to confirm that it is of an acceptable scientific standard.

# Reviewer report: Reviewer response for version 2

**DOI:** 10.5256/f1000research.174399.r341410

**Published:** 2024-12-13

**Authors:** Deepa Handu

**Affiliations:** 1 Academy of Nutrition and Dietetics, Chicago, Illinois, USA

Thank you for addressing the comments I had suggested in initial review.

Are the rationale for, and objectives of, the Systematic Review clearly stated?

Yes

Is the statistical analysis and its interpretation appropriate?

Partly

If this is a Living Systematic Review, is the ‘living’ method appropriate and is the search schedule clearly defined and justified? (‘Living Systematic Review’ or a variation of this term should be included in the title.)

No

Are sufficient details of the methods and analysis provided to allow replication by others?

Partly

Are the conclusions drawn adequately supported by the results presented in the review?

Partly

Reviewer Expertise:

evidence based methodologists, systematic review, scoping review, nutrition, public health, etc

I confirm that I have read this submission and believe that I have an appropriate level of expertise to confirm that it is of an acceptable scientific standard.

# Reviewer report: Reviewer response for version 1

**DOI:** 10.5256/f1000research.164365.r288622

**Published:** 2024-09-23

**Authors:** Palak Gupta

**Affiliations:** 1 Utah State University, Logan, UT, USA

Thank you for the opportunity to review this article. I appreciate the effort put into this work. However, I have a few comments regarding the methods and conclusions.

 Methods:

 Was this scoping review registered anywhere (e.g., PROSPERO)?

 The eligibility criteria for inclusion need further clarification. How were the articles screened, and who was responsible for the screening process? This should be clearly outlined to ensure transparency.

 the methodology could benefit from clarification on how comprehensiveness was ensured. Researchers started with 49 and narrowed to 7 policies. Were there any search terms or Boolean logic used? Did the researchers consult any experts to validate that all relevant ministries and policies were included? 

 the decision to exclude policies focused on micronutrient deficiencies and wasting/stunting could be elaborated upon, given the connection between malnutrition and obesity. A more detailed rationale for exclusion would strengthen this section. Also, clarifying whether this exclusion was based on a theoretical framework, or stakeholder consultation would add strengthen this section. 

 Conclusion: 

 The conclusion can be strengthened- How your findings contribute to your overarching goals?

 To create a stronger conclusion, consider adding a call-to-action directed at policymakers.

Are the rationale for, and objectives of, the Systematic Review clearly stated?

Yes

Is the statistical analysis and its interpretation appropriate?

Partly

If this is a Living Systematic Review, is the ‘living’ method appropriate and is the search schedule clearly defined and justified? (‘Living Systematic Review’ or a variation of this term should be included in the title.)

Not applicable

Are sufficient details of the methods and analysis provided to allow replication by others?

Partly

Are the conclusions drawn adequately supported by the results presented in the review?

Partly

Reviewer Expertise:

Food insecurity, hunger, Nutrition, public health

I confirm that I have read this submission and believe that I have an appropriate level of expertise to confirm that it is of an acceptable scientific standard, however I have significant reservations, as outlined above.

# Response

**Published:** 2024-11-04

**Authors:** Shalini Bassi

**Affiliations:** Public Health Foundation of India, India

**Response Document-**

**Methods:**

1. Was this scoping review registered anywhere (e.g., PROSPERO)?

**Response: **Thank you for your insightful comment regarding the registration of our scoping review. In our case,  the scoping review was not been registered anywhere, since our research was primarily exploratory and unlike systematic review did not assess the impact of any intervention, we did not feel the need for registration. Secondly, by the time we considered registering, we had already made significant progress in terms of defining the research question and developing the protocol.  However, we acknowledge that registering a scoping review is a valuable step to increase transparency and avoid duplication of efforts and will definitely be considered henceforth.

  

1. The eligibility criteria for inclusion need further clarification. How were the articles screened, and who was responsible for the screening process? This should be clearly outlined to ensure transparency.

**Response:** Thank you for your question. We have elaborated the search strategy as per your suggestion. The polices were screened based on eligibility criteria which have been clearly re-defined. Two researchers (NT, DB) conducted the screening process and any discrepancies were resolved through discussion or consultation with a third researcher (SB) to ensure consistency and minimize bias. All have been included in the strategy.

  

1. The methodology could benefit from clarification on how comprehensiveness was ensured. Researchers started with 49 and narrowed to 7 policies. Were there any search terms or Boolean logic used? Did the researchers consult any experts to validate that all relevant ministries and policies were included?

**Response- **Thank you for your question.  We have edited the search strategy, to provide a more detailed account of the process, for improved clarity as per your suggestion. In addition, we consulted domain experts to ensure that all pertinent ministries and policies were considered to validate the comprehensiveness of our search.

  

1. The decision to exclude policies focused on micronutrient deficiencies and wasting/stunting could be elaborated upon, given the connection between malnutrition and obesity. A more detailed rationale for exclusion would strengthen this section. Also, clarifying whether this exclusion was based on a theoretical framework, or stakeholder consultation would add strengthen this section.

**Response: **Thank you for your suggestions **. **The decision to exclude policies was based on the specific focus of the review which was on overweight and obesity. While malnutrition in all forms is interconnected, the causes, interventions and policies for undernutrition (including micronutrient deficiencies and wasting/stunting) differ significantly from those targeting obesity. Hence, as per our understanding, the inclusion of these would have diluted the reviews focus.  Additionally, this decision was also supported by domain experts. We have added this to the manuscript as well

 Conclusion:

1. The conclusion can be strengthened- How your findings contribute to your overarching goals?

**Response-** Thank you! We have revised the conclusion based on your suggestion.

# Reviewer report: Reviewer response for version 1

**DOI:** 10.5256/f1000research.164365.r303344

**Published:** 2024-09-03

**Authors:** Deepa Handu

**Affiliations:** 1 Academy of Nutrition and Dietetics, Chicago, Illinois, USA

Thank you for the opportunity to review this article.

 I have two major comments for this paper:

 -Rigor of Methods for Scoping Review: Though the authors state that this is a "Scoping Review" and follows Arksey and O’Malley’s steps of conducting a scoping review……………each step of this process is not conducted rigorously and does not meet the methods standard for scoping reviews.  

 - Also, scoping reviews are conducted to see what is the availability of evidence on a certain topics………….and secondly- how are these findings going to help your future actions?  OR how the findings of this scoping review guide your next steps.

 - the rationale for conducting a scoping review should be strong.

 - Please link of your findings to stated research questions/objectives.

 Search strategy needs more details.  Did the authors just hand search government files or is there any database of government documents available.  Sharing a search strategy is a main component of scoping reviews.

 Seems like most of their search is “grey literature”- if yes then that should be clearly stated and what other sources of grey literature did they search. How do reviewers confirm they conducted an exhaustive search.

  A PRISMA flow chart of search should be provided. Also, what were the search dates for these article/policies to be included?

 Was this scoping review registered anywhere??

 Eligibility criteria: how were the articles screened and who screened the articles for inclusion.

 Most of my comments are related to the rigor of the methods, transparency of reporting, and how will the findings contribute to next steps.

 Would suggest that authors refer to PRISMA Scoping Review checklist to help address all the key points necessary for a Scoping review.

Are the rationale for, and objectives of, the Systematic Review clearly stated?

Yes

Is the statistical analysis and its interpretation appropriate?

Partly

If this is a Living Systematic Review, is the ‘living’ method appropriate and is the search schedule clearly defined and justified? (‘Living Systematic Review’ or a variation of this term should be included in the title.)

No

Are sufficient details of the methods and analysis provided to allow replication by others?

Partly

Are the conclusions drawn adequately supported by the results presented in the review?

Partly

Reviewer Expertise:

evidence based methodologists, systematic review, scoping review, nutrition, public health, etc

I confirm that I have read this submission and believe that I have an appropriate level of expertise to confirm that it is of an acceptable scientific standard, however I have significant reservations, as outlined above.

# Response

**Published:** 2024-11-04

**Authors:** Shalini Bassi

**Affiliations:** Public Health Foundation of India, India

**Response Document-**

 1. Rigor of Methods for Scoping Review: Though the authors state that this is a "Scoping Review" and follows Arksey and O’Malley’s steps of conducting a scoping review……………each step of this process is not conducted rigorously and does not meet the methods standard for scoping reviews. 

**Response:** Thank you for this observation. We have revised the manuscript and detailed description of each step of the scoping review process aligning with the Arksey and O’Malley framework in the methodology and result section added.

 2. Also, scoping reviews are conducted to see what is the availability of evidence on a certain topics………….and secondly- how are these findings going to help your future actions?  OR how the findings of this scoping review guide your next steps. The rationale for conducting a scoping review should be strong. Please link of your findings to stated research questions/objectives.

**Response: **Thank you! The scoping review  map the availability of evidence on how SBCC is integrated into policies on overweight obesity in India. The findings will guide future actions by identifying gaps, informing and suggestion mid-course correction and policy implications. As suggested, the same has been mentioned in the introduction for clarity. Further on as per your suggestion, we have linked the findings to the research questions.

 3. Search strategy needs more details.  Did the authors just hand search government files or is there any database of government documents available.  Sharing a search strategy is a main component of scoping reviews.

**Response: **Thank you! We have edited the search strategy, to provide a more detailed description of the process, enhancing clarity based on your suggestion.

  

- Seems like most of their search is “grey literature”- if yes then that should be clearly stated and what other sources of grey literature did they search. How do reviewers confirm they conducted an exhaustive search.

**Response- **Thank you for your valid suggestion. We have edited the search strategy to mention grey literature

  

- A PRISMA flow chart of search should be provided. Also, what were the search dates for these article/policies to be included?

**Response:** Thank you! As suggested a revised PRISMA flow chart of the search strategy has been attached with the manuscript as Figure 1.  Additionally, the search dates of the policy instruments [1] is already been included in the manuscript.

*“The search was conducted by researchers between March-June 2023.”*

 4. Was this scoping review registered anywhere?

**Response: **Thank you for your insightful comment regarding the registration of our scoping review. In our case, the scoping review was not registered anywhere, since our research was primarily exploratory and unlike systematic reviews did not look to assess the impact of any intervention, hence we did not feel the need for registration. Secondly, by the time we considered registering, we had already made significant progress in terms of defining the research question and developing the protocol. Thirdly, research questions and methodologies evolved significantly during the review process, this would have complicated the adherence to any prior registration plans. However, we acknowledge that registering a scoping review is a valuable step to increase transparency and avoid duplication of efforts and will definitely be considered henceforth.

 5. Eligibility criteria: How were the articles screened and who screened the articles for inclusion.

**Response: **Thank you for the question. We have elaborated the search strategy as per your suggestion. The policies were screened based on eligibility criteria. Two researchers (NT, DB) conducted the screening process and any discrepancies were resolved through discussion or consultation with a third researcher (SB) to ensure consistency and minimize bias. All have been included in the strategy.

[1] Policy instrument’s throughout the paper has been used to cover programmes, schemes, regulations and guidelines
